# Discussion to: Pacemaker implantation following tricuspid valve annuloplasty

**DOI:** 10.1016/j.xjon.2023.09.021

**Published:** 2023-09-22

See Article page 276.

Presenter: Dr Sigurdur Ragnarsson

**Dr Anelechi Anyanwu***(New York, NY)*. Thank you. Thanks. Great presentation. I have 4 questions. My first 1 you've probably just answered from limitations. You didn't state the indications for the tricuspid annuloplasty or the proportion of patients who had severe tricuspid regurgitation (TR) so it seems you don't have that because I was going to ask you whether the pacemaker rates were different for those when the ring was placed for severe TR as opposed to prophylactic, but you don't know.

**Figure:**
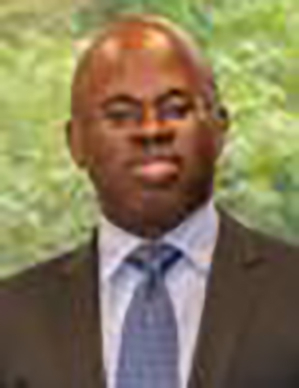

**Dr Sigurdur Ragnarsson***(Lund, Sweden)*. I think this is an excellent point. And no, so the dataset did not include the risk, the severity of TR. However, we know that only 15% of patients undergoing mitral valve surgery during this study period had concomitant tricuspid repair, which sort of indicates that it's an underused modality, and thus I think almost everyone in the cohort had severe TR.

**Figure:**
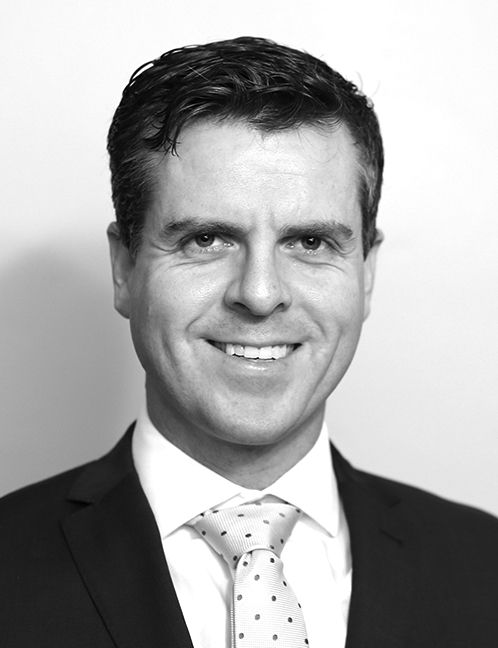

**Dr Anyanwu**. So, my second question hopefully you can answer. From the data you showed, two-thirds of your patients had atrial fibrillation but only one-third had an ablation. So, it makes one wonder whether there could have been an influence of either residual atrial fibrillation or the procedures used to treat atrial fibrillation. So, let's say you took out everyone who had atrial fibrillation from your study. What was the pacemaker rate in those left behind?

**Dr Ragnarsson**. Yes. Excellent question. It was actually exactly the same, 14%. And the reason is that patients who had ablation done had a higher risk of pacemaker compared to patients with no prior atrial fibrillation, whereas patients who did not have ablation had a lower risk of pacemaker compared to patients with no prior history of atrial fibrillation.

**Dr Anyanwu**. Seems that 14% is becoming a magic number for pacemakers.

**Dr Ragnarsson**. Yeah, it's a magic number, yeah.

**Dr Anyanwu**. Okay. So, there's a vast area of data that links chronic right ventricle at pace into adverse outcomes in terms of heart failure and survival. Indeed today, I don't know whether you have the lunchtime session, but Iribarne presented data from the Cardiothoracic Surgery Network study where they looked at more than 32,000 patients from New York and California who had tricuspid annuloplasty. They also found 14%. That's why I say 14% is a magic number. But 1 thing they did find is that permanent pacemakers [inaudible] were survival, and the curves continued to diverge after 10 years, but you actually found better survival in patients who got pacemakers and that's something that really doesn't exist in the literature. So, it makes me wonder whether either you are putting pacemakers in patients who subsequently are not being paced in the long-term; that is, they did not need it, or there's a problem with your data—either with miscoding or the follow-up of variables. So, it takes me to my question, which is, do you know what the indications—in Sweden, what's the practice in terms of who gets a pacemaker after heart surgery when you decide to put it in and the triggers?

**Dr Ragnarsson**. So, in regard to the quality of data, I would say that the survival data is absolutely pristine and the data on who gets the pacemaker is also very reliable. In regard to the indication and timing of pacemaker implantation following cardiac surgery, that's institutional practice. We have not looked into whether the median time from surgery to implantation varies between centers. That's something that we could do. But I cannot really talk much about institutional practice in the different centers.

**Dr Anyanwu**. How about in your center, in your experience?

**Dr Ragnarsson**. In my experience, we usually wait 3 to 4 days at least before contacting electrophysiology, and then we'd put a pacemaker—

**Dr Anyanwu**. And when you see the patient a month or 2 months later, are they generally still placing or not placing?

**Dr Ragnarsson**. So, I can't really—I don't know the answer. I do think that we can investigate though, with data from the Swedish Pacemaker Registry. So, this is something that I will take up with my group.

**Dr Anyanwu**. Okay, so my last question really is because I'm going back to New York tonight. So, what's my take-home message? Because we're going to have patients, we're going to be putting these tricuspid rings in, and now I'm worried I've got to tell them that they have a 15% pacemaker risk. So, well, are there patients in which I should, based on your analysis, for example, you found some risk factors for pacemaker, so are there patients that I should avoid doing a prophylactic tricuspid repair or any tricuspid repair? And are there any surgical techniques or medical interventions that I should apply to people at risk? How should I change my practice as a result of your study?

**Dr Ragnarsson**. Well, I don't think that you should personally change your practice because the rate is 2.5% at your institution. However, I think we can definitely take a look at surgical techniques and try to reduce the rate of pacemaker implementation in Sweden. I do not think that these numbers should scare us. I think we should be doing more tricuspid valve annuloplasties, and we should also not be discouraged if our patient does need a pacemaker following tricuspid valve repair.

**Dr Anyanwu**. Sorry, since you mentioned my institution, you're forced to get a fifth question. Why do you think—why do you think it's 2.5% in 1 institution and 14% in another? What do you think the difference is?

**Dr Ragnarsson**. I think—

**Dr Anyanwu**. Do you believe it's really 2.5% or do you think—go ahead.

**Dr Ragnarsson**. No, I do. Yeah. Ooh, you're putting me in a tough spot. I think—I think it's the volume, volume, volume.

**Dr Anyanwu**. Yeah, but you do thousands in Sweden, so you can achieve the same. They do 30,000 in New York and California, where it's 14%. So why is 2% in 1 and 14% in another, what do you think?

**Dr Ragnarsson**. So first of all, this data is not really comparable to patients that get tricuspid valve repair for moderate TR or dilated annulus because this is most likely sicker patients in my baseline—

**Unidentified Speaker 1**. We have a quick answer—1 quick question, Ani—

**Dr Anyanwu**. Thank you so much.

**Unidentified Speaker 1**. Thank you. Because we're going to stay on time. Quick question, quick answer, and I'm making 1 comment. Very quick.

**Unidentified Speaker 2**. All right, so quick. [inaudible] Poland. Quick question is, how about endocarditis? Because if it is low risk, it may explain the protective effect for the better survival when you have got pacemaker.

**Unidentified Speaker 1**. Okay, quick answer.

**Dr Ragnarsson**. We have not looked at endocarditis following pacemaker, but that's something that can be extracted from the dataset.

**Unidentified Speaker 1**. Okay, so I'm going to make 1 comment, and we'll stop. Thank you very much for your presentation. I'm going to invoke the Tirone David rule. We can blame the patient and blame the pacemaker or examine ourselves. Because I know that our institution, it is 2.5%, and it's state-reported data. In New York State, it's state reported so it's all adjudicated, so we know what it is. We've looked now at over, I don't know, 2000 patients. We just haven't published it yet, but our publication in 2015 was 748, it's 2.5%. Steve, I know your group is the same numbers, 5%, 6%, many 5%. So, this idea that it's 14% is only based on our responsibility as surgeons. We need to put the sutures in correctly. We need to change the kinds of bands or techniques we use. Who knows what we need to do, but you just cannot say that it's 14%. It's 14% in the real world, across all volumes and all centers, I guess, but this is a pretty learnable technique, believe me. This is not the hardest thing anyone in any of those centers does every week, and we've got to do better for our patients with tricuspid valve repair. And we just have to share the ways we do it amongst each other, and we have to move this field forward. We cannot talk about 14% pacemaker rates for the next 5 years. We need to evolve just like Tirone did. Thank you very much.

**Dr Ragnarsson**. Thank you very much, Dr Adams. [applause]

## Conflict of Interest Statement

The authors reported no conflicts of interest.

The Journal policy requires editors and reviewers to disclose conflicts of interest and to decline handling manuscripts for which they may have a conflict of interest. The editors and reviewers of this article have no conflicts of interest.

